# Developing a Framework and Electronic Tool for Communicating Diagnostic Uncertainty in Primary Care: A Qualitative Study

**DOI:** 10.1001/jamanetworkopen.2023.2218

**Published:** 2023-03-09

**Authors:** Maram Khazen, Maria Mirica, Narath Carlile, Alissa Groisser, Gordon D. Schiff

**Affiliations:** 1 Department of Health Systems Management, Harvard Medical School and Brigham and Women’s Hospital, Boston, Massachusetts; 2 Now with Max Stern Yezreel Valley College, Yezreel Valle, Israel; 3 Department of Medicine, Division of General Medicine Center for Patient Research and Practice, Brigham and Women’s Hospital, Boston, Massachusetts; 4 Department of Pediatrics, Children’s National Hospital, Washington, DC; 5 Center for Patient Safety Research and Practice, Division of General Internal Medicine, Brigham and Women’s Hospital, Boston, Massachusetts; 6 Harvard Medical School Center for Primary Care, Boston, Massachusetts; 7 Center for Primary Care, Harvard Medical School, Boston, Massachusetts

## Abstract

**Importance:**

Communication of information has emerged as a critical component of diagnostic quality. Communication of diagnostic uncertainty represents a key but inadequately examined element of diagnosis.

**Objective:**

To identify key elements facilitating understanding and managing diagnostic uncertainty, examine optimal ways to convey uncertainty to patients, and develop and test a novel tool to communicate diagnostic uncertainty in actual clinical encounters.

**Design, Setting, and Participants:**

A 5-stage qualitative study was performed between July 2018 and April 2020, at an academic primary care clinic in Boston, Massachusetts, with a convenience sample of 24 primary care physicians (PCPs), 40 patients, and 5 informatics and quality/safety experts. First, a literature review and panel discussion with PCPs were conducted and 4 clinical vignettes of typical diagnostic uncertainty scenarios were developed. Second, these scenarios were tested during think-aloud simulated encounters with expert PCPs to iteratively draft a patient leaflet and a clinician guide. Third, the leaflet content was evaluated with 3 patient focus groups. Fourth, additional feedback was obtained from PCPs and informatics experts to iteratively redesign the leaflet content and workflow. Fifth, the refined leaflet was integrated into an electronic health record voice-enabled dictation template that was tested by 2 PCPs during 15 patient encounters for new diagnostic problems. Data were thematically analyzed using qualitative analysis software.

**Main Outcomes and Measures:**

Perceptions and testing of content, feasibility, usability, and satisfaction with a prototype tool for communicating diagnostic uncertainty to patients.

**Results:**

Overall, 69 participants were interviewed. A clinician guide and a diagnostic uncertainty communication tool were developed based on the PCP interviews and patient feedback. The optimal tool requirements included 6 key domains: most likely diagnosis, follow-up plan, test limitations, expected improvement, contact information, and space for patient input. Patient feedback on the leaflet was iteratively incorporated into 4 successive versions, culminating in a successfully piloted prototype tool as an end-of-visit voice recognition dictation template with high levels of patient satisfaction for 15 patients with whom the tool was tested.

**Conclusions and Relevance:**

In this qualitative study, a diagnostic uncertainty communication tool was successfully designed and implemented during clinical encounters. The tool demonstrated good workflow integration and patient satisfaction.

## Key Points

**Question:**

What are clinician and patient perspectives and innovative ways to communicate diagnostic uncertainty to patients?

**Findings:**

In this qualitative study, interviews with 69 primary care physicians, patients, and informatics and quality/safety experts suggested key features for a diagnostic uncertainty communication tool include need to sensitively acknowledge uncertainty, promote patient engagement, consider clinician’s workflow, and balance verbal conversation while producing a printed patient handout. Elements of the iteratively developed voice-enabled tool included most likely diagnosis, follow-up plan, test limitations, expected improvement course, and contact information.

**Meaning:**

In this qualitative study, a prototype of a diagnostic uncertainty communication tool was successfully conceptualized, designed, and piloted.

## Teaser

This qualitative study describes the development of a tool to audio record the clinician and patient interaction during a patient encounter to communicate diagnostic uncertainty.

## Introduction

Diagnostic quality, as defined by the National Academy of Medicine Committee on Diagnostic Error in Healthcare, relies on effective communication.^1^ One key aspect of meaningful diagnostic communication and minimizing diagnostic errors is conveying diagnostic uncertainties to patients during clinical visits.^2,3,4,5^ However, there has been a paucity of studies and interventions addressing ways to best manage this critical element for conversing with patients regarding uncertainties in diagnosis.^6,7^

The current state of the art regarding managing diagnostic uncertainty is both underdeveloped and contentious.^8,9^ Some have suggested that the absence of direct utterances of uncertainty or indirect expressions of imperfect knowledge reflects clinician unwillingness to acknowledge uncertainty.^10,11^ Medical training offers little guidance on how best to develop communication skills to convey uncertainty.^12,13^ Many fear that in expressing uncertainty clinicians risk being perceived by patients as less knowledgeable, confident, or competent.^14,15,16^ Nonetheless, inappropriate management of diagnostic uncertainty could result in either diagnostic delays (if patients are falsely reassured and do not perceive a need to consider alternative diagnoses) or unnecessary anxiety (if nonreassuring communication overly worries them).^17,18^

Emerging evidence, as well as ethical standards respecting patient autonomy,^19^ show that acknowledging diagnostic uncertainty has potential benefits, including strengthening and fostering clinician-patient relationships and trust, promoting better patient engagement, and advancing shared decision-making.^20,21^ Patients who come to clinicians for reassurance (there is nothing serious) or a definitive explanation (your diagnosis is X) will not likely welcome what they might perceive as equivocal, vague, hedged, or evasive answers from physicians appearing to lack confidence, knowledge, and certainty. Yet honesty and transparency demand that clinicians more modestly step away from an arrogant overconfidence and candidly and accurately acknowledge what they do and do not know about the diagnosis. This transparency is a prerequisite to promoting shared decision-making and ensuring safe action plans, monitoring, and follow-up.

As part of a project funded by the Controlled Risk Insurance Company (CRICO), Harvard Hospitals’ malpractice insurer, we conducted a study examining best practices to communicate and manage diagnostic uncertainty. We developed an innovative prototype of a communication tool that included (1) a clinician’s guide of best practices to managing diagnostic uncertainty, (2) a paper-based patient leaflet, and (3) an electronic prototype of the leaflet incorporated in the institutional electronic health record. We pilot tested this tool during clinical encounters and collected feedback to understand how implementation of the tool was perceived by patients.

Thus, we examined the following research questions: (1) What are the key elements as perceived by clinicians and patients that would facilitate understanding and communicating diagnostic uncertainty? (2) What are approaches and the optimal language clinicians can use to convey uncertainty to patients? (3) How can clinicians best incorporate these elements into their clinical workflow? (4) How can these elements be incorporated into a patient-specific communication tool that clinicians can practically implement during the encounter to produce a written diagnosis end-of-visit summary?

## Methods

### Study Design

We used an exploratory descriptive qualitative research design.^22^ Our approach was constructivist,^23^ using the personal experiences of PCPs and informatics experts and acquired knowledge and in-depth learning from patient experiences regarding how clinicians communicate diagnostic uncertainty and what are the best ways to manage this uncertainty. The study took place between July 2018 and April 2020 and was approved by the Mass General Brigham Institutional Review Board. We began by reviewing published literature regarding diagnostic uncertainty communication. We then used interviews and focus groups with patients, informatics experts, and clinicians to develop a prototype communication tool. Given the diversity of multidisciplinary sources in our convenience samples of (eg, clinicians, patient advocates, quality experts) we did not collect details of participant demographic characteristics. All participating patients and clinicians provided verbal informed consent as specified by the approving institutional review board. No financial compensation was provided to participants. The study was conducted in 5 phases accompanied by an iteratively revised patient leaflet and a clinician guide to managing diagnostic uncertainty (eAppendix 1 in Supplement 1). This study followed the Standards for Reporting Qualitative Research (SRQR) reporting guideline.

#### Phase 1

During this background phase, the principal investigator (G.D.S.) and a research assistant (A.G.) reviewed literature and conducted a panel discussion with PCPs and communication experts (n = 5). Based on the findings from this stage, 4 clinical vignettes were developed. The vignettes described the following general medical diagnostic uncertainty scenarios: (1) enlarged lymph node in a patient with a history of lymphoma in remission (ie, worrisome for a recurrence), (2) new-onset headache, (3) fever and upper respiratory infection, and (4) subacute low back pain (eAppendix 2 in Supplement 1).

#### Phase 2

The study team identified and invited a convenience sample of expert PCPs at the research institution and nationwide for one-on-one interviews. During the interviews, these PCPs were presented with the 4 clinical vignettes and asked to think aloud, reflecting on ways they might typically discuss diagnoses and convey uncertainty to such a patient. To prompt different perspectives and divergent communicative styles, the degree of uncertainty was calibrated so the first case presented less diagnostic uncertainty but was more worrisome and urgent, whereas the other cases posed varying degrees of urgency, seriousness, and diagnostic uncertainty. The principal investigator role-played the patient to simulate a medical encounter. Interviews lasted 30 to 60 minutes and were audio recorded and transcribed. Based on these interviews, we developed the initial draft of a patient leaflet and an accompanying clinician guide to communicating uncertainty. (eAppendix 1 in Supplement 1).

#### Phase 3

The research team conducted 3 focus groups with patients who represented exemplary local and national patient organizations (Society to Improve Diagnosis in Medicine Patient Engagement Committee, Beth Israel Hospital Patient and Family Relations Committee, and Medically Induced Trauma Support Services Patient Board). Each focus group consisted of 6 to 10 participants (total 25; 21 women), lasted 60 to 80 minutes, and solicited feedback about the patient leaflet and the clinician guide. Trigger questions included (1) What is your general reaction to the leaflet? (2) How would you feel about receiving such a leaflet? (3) Do you have any suggestions for how to revise and use the leaflet? Many of the focus group participants had previously worked together across their organizations and likely had a level of comfort with each other that facilitated the conversation. Their feedback helped segment the content of the patient leaflet into 6 key domains of communicating diagnostic uncertainty. At this stage, we developed the next version of the diagnostic uncertainty communication tool that included a draft of the clinician guide and a patient leaflet with a fillable template.

#### Phase 4

The draft leaflet was then further reviewed during 13 one-on-one interviews (6 by phone, 7 face-to-face) with 8 (4 women) practicing PCPs and 5 (1 woman) informatics and communication experts to solicit further input into the leaflet content and design, as well as suggestions regarding how to best incorporate the leaflet into the clinical encounter. Interviews lasted 30 to 40 minutes and were audio recorded and transcribed. Based on analysis of these interviews, the leaflet was finalized as a 1-page document that was converted to a fillable electronic health record template with prompts for the 6 key domains of communicating diagnostic uncertainty.

Functions that the final version of the tool was designed to perform included the ability (1) to be dictated during a patient visit, (2) to be printed as a handout and incorporated into the electronic health record after visit summary, and (3) to serve as a tool for capturing the clinician’s assessment in the clinical note.^24,25^

#### Phase 5

Two PCPs (N.C., G.D.S.) involved in developing the patient leaflet prototype tested it with a convenience sample of 15 (9 women) patients during primary care encounters if patient symptoms raised diagnostic uncertainty. As part of their conversation with the patients, the physicians dictated into voice recognition software (Epic Dragon One; Dragon Medical Dictation Software) using a dictation microphone (PowerMic III; Nuance) that facilitated rapid navigation to the templated fields. The physicians were instructed to look directly at patients while dictating and address each element in the templated leaflet, pausing at various points to allow the patients to contribute additional information. At the end of each visit, the templated leaflet was printed. A communications researcher (M.K.) then interviewed patients to solicit their feedback on the experience, including content of the leaflet and visit process related to diagnostic communication. Patients were asked to reflect on preferences between dictating the after-visit summary via voice recognition vs typing, what elements of the leaflet/after-visit summary content they liked vs did not understand or like, how well this summary captured what went on in the encounter, and whether they would like to have similar diagnostic summaries in the future. Feedback from these interviews provided insights regarding key elements from the patient’s perspective to better understand the diagnosis and follow-up plan.

### Statistical Analysis

Interview and focus group data were transcribed verbatim and analyzed by the team members (M.K., M.M., G.D.S.). Deductive and inductive analytic approaches to coding were implemented using NVivo, version 10 software (Lumivero).^26^ First, the data were tagged and organized according to the topics that mapped to the a priori–developed research questions (eg, key elements and techniques for communicating about uncertainty, integrating these elements into the workflow, key elements facilitating understanding the diagnosis and uncertainties). Second, emergent codes were developed based on the participants’ responses (eg, the patient leaflet capturing the essence of the encounter, conversational mode of dictating leaflet). This coding strategy of mixing of concept-based and data-based codes enabled the research team to organize the responses by topic while ensuring the codes were grouped together based on the data, moving from descriptive themes to more analytic categories.^26,27^ In addition, one of us (M.K.) thematically analyzed the data by identifying recurring themes.^28,29^ To ensure consistency, the research team (M.K., M.M., G.S.) met weekly to discuss new codes, their definitions, and fit with the entire data set. Researchers reached full agreement on coding decisions after these coding reconciliation meetings.

## Results

Findings from each of the phases of the project, including how each phase informed the subsequent one, are summarized in Table 1. The phase 1 sample included 11 PCPs (5 women). Two categories emerged from data analyses of interviews and focus groups: requirements for developing a communicating diagnostic uncertainty tool and main domains of the diagnostic uncertainty tool. In addition, the first 3 phases of the study distilled a clinician guide focusing on the best ways to communicate uncertainty.

**Table 1.** Patient Leaflet Development and Implementation Process

Phase	Methods	What was learned	What emerged and was developed
1	Background work: literature review and panel discussion	Paucity of strategies addressing diagnostic uncertainty during clinical encounters and helpful language for managing uncertainty in clinical contexts Communication strategies need to be mindful and respectful of clinical workflows	4 Scenarios with embedded diagnostic uncertainty were developed to probe and develop language guidance
2	Capturing language used by experienced, PCPs via personal think-aloud interviews via 4 scenarios	Main themes: Importance of patient engagement in the diagnostic process and transparent communication Need to provide supportive/validating explanations and reassurance to anxious patients Explaining in lay terms the differential diagnosis, dynamic nature of disease, and monitoring symptoms over time (the test of time)	Version I of patient leaflet—a 2-page handout explaining diagnostic uncertainty in general (side 1) and information on patient’s specific diagnosis, differential, and follow-up (side 2) along with accompanying clinician guide
3	Three 60- to 80-min structured focus groups with 25 patients to solicit feedback on draft version I of leaflet and clinician guide	Main themes: Leaflet should be shorter; mixed support for general educational explanation of diagnostic uncertainty; some felt it represented more evasive legal caveats rather than intended honest transparency Concerns leaflet might substitute for clinician-patient conversation about uncertainty, which patients preferred to happen verbally Patients especially appreciated language about working together and explicit invitation for partnership to coproduce the diagnosis Some preferred structured rather than narrative leaflet, with boxed sections instead of free text format of version I	Clinician guide revised and version II of the leaflet was produced (a 2-page templated leaflet); feedback improved formatting, content, and perceived leaflet utility; leaflet contained briefer educational information about diagnostic uncertainty and reinforced clinician-patient partnership language
			A leading patient safety advocate drafted alternate 1-page version illustrating conceptual and design approaches (version III, incorporating selected features of versions I and II)
4	Version III tested via interviews with 8 PCPs and 5 medical informatics experts	Main themes: Importance of addressing workflow concerns, workloads, and limited encounter time Producing a timely leaflet for handing out at end of visit is challenging; voice recognition software proposed to facilitate this individualized leaflet creation during the visit Language in leaflet could serve multiple purposes: (1) populating the note assessment, (2) end-of-visit summary, (3) verbal communication of clinicians’ diagnostic assessment during the encounter Reiterated need for leaflet to emphasize the clinician-patient partnership and working together theme	Leaflet was shortened to 1 page and focused on actionable steps for patients toward a more definitive diagnosis; general uncertainty education text trimmed
			Version IV iteratively designed and debugged using innovative Epic Dragon One/EHR template and voice recognition technology (using Nuance PowerMic III) to dictate and print as part of the end-of-visit summary patient handout
5	Two PCPs implemented version IV during 15 encounters with patients with acute diagnostic concerns; patients interviewed immediately after the visit to solicit views on process and leaflet	Main themes Dictating the templated tool during the encounter was perceived by patients as a personal conversation, despite the fact that the PCP was dictating into the microphone Patients strongly liked integrating the leaflet into the end-of-visit summary printout and said it accurately captured what was said during the encounter Implementing the dictated template served as a helpful forcing function for PCPs to incorporate key diagnostic safety and uncertainty elements Despite hopes it could serve multiple purposes, it was challenging to achieve the right language for the patient leaflet to simultaneously serve as text for populating the clinical note along with leaflet	Final prototype refined and successfully used by committed test PCPs; patients gave glowing reviews of appreciation of the concept and of the leaflet they received
			Process/leaflet seemed applicable to only selected encounters (ie, was a new diagnostic issue being evaluated); unclear if less committed PCPs would readily adopt; patient leaflet did not work as a substitute for the clinical note assessment
			Dictating leaflet via the voice recognition software was perceived by patients as effectively serving as personalized verbal explanation of their problem

Abbreviations: EHR, electronic health record; PCP, primary care physician.

### Clinician Guide

The clinician guide summarized educational teaching points giving tips on optimal ways to communicate diagnostic uncertainty. It identified 4 main guiding principles: (1) validating patient experiences and symptoms, (2) transparency about uncertainty, (3) creating a concrete plan, and (4) not assuming patient worries and not diminishing their symptoms (eAppendix 1 in Supplement 1).

### Requirements for Communicating Diagnostic Uncertainty Communication Tool

Five major themes emerged regarding requirements of developing an effective communication uncertainty tool. The tool would need to (1) acknowledge desirability of communicating uncertainty, (2) promote patient’s engagement, (3) fit into clinician’s workflow and time constraints, (4) balance conversational style and manage workloads, and (5) produce a printed leaflet reflecting the clinical conversation that could be handed to patients at the end of the visit. A summary of the findings is presented in Table 2.

**Table 2.** Key Elements Shaping the Content and Implementation of the Patient Leaflet Version IV

Main element	Example: selected quotations	Source/phase of the study
Acknowledging and communicating uncertainty	Make sure that unstated things are discussed. So, if you see uncertainty in a patient you have to sanction it. Patients need to feel comfortable; otherwise, they think they are challenging your judgment.	PCPs/phase 2
	It is critical that people understand it is not a definite situation, that there are several variables around uncertainty.	Patient focus group/phase 3
	Most patients understand that doctors don’t always have all the answers, and if you pretend that you do, then you’re not being as transparent, and you should work to be more transparent.	PCP/phase 4
	I see myself saying this to the patient [referring to the first sentence in the leaflet: diagnosis is not 100% certain] that your situation is uncertain.	
Workflow concerns	I think that people are generally moving away from anything that is handwritten so they would do it as a form template to make it easier to integrate in the note.	Informatics experts/phase 4
	I understand it is more work for the doctor. It is more questions. But maybe it is not because the doctor anyway is asking the questions, but this is a record for the patient.	Patient/phase 5
Patient’s engagement	Talking about uncertainty is a moral obligation, if I am engaging in shared decision-making and I am sure there is uncertainty, and I am asking the patient to be a partner. I need them to know that I am not certain about things.	PCP/phase 2
	I think that some…want to be told what to do. But other patients are going there because they want to have that engagement, or they want to feel like they’re being heard.	PCP/phase 4
	When there is uncertainty, patients should be more active and engaged in the dialogue.	Patient/phase 3
	I think it [referring to version IV of leaflet] is really helpful. It gives me more information, so it is helpful for me to be engaged.	Patient/phase 5
Conversational workflow can also be captured in written leaflet	I think typically what I do is a much more than conversation. And so, I’m engaging. I try to stop every 4 or 5 seconds [to ask] what questions [they have]?	PCP/phase 2
	Something like this [referring to version II] can be more effective in a dialogue instead of a piece of paper.	Informatics experts/phase 4
	I would like to have it as a dialogue. The document as written [referring to version II], I do not think the patient will read it.	Patient/phase 3
	If you want the doctor to communicate thoughts and processes, the easier and fastest way to do that is to talk to the patient.	
	He [doctor using voice recognition template] stated something and stopped and then if I had something to add he definitely stopped and addressed it. So, I thought it was kind of a conversation.	Patient/phase 5
Template capturing the encounter to overcome workflow concerns	A template would be better if this goes into the note and not as something added that the doctor has to do.	PCP/phase 4
	You need a [customizable narrative to populate the] template, not something generic. Something that could work for each patient.	Patient/phase 3
	This is something patients take with them. Sometimes I feel that I cannot take in the whole information and later this will be a good reference tool.	Patient/phase 5

Abbreviation: PCP, primary care physician.

### Main Domains of Communicating Diagnostic Uncertainty Tool

Literature review revealed a paucity of evidence supporting any approach or language for communicating diagnostic uncertainty to patients. Thus, we iteratively developed a new framework from various commentaries and recommendations that we successively refined with a series of expert interviews. The framework (Table 3) used to design a tool to communicate diagnostic uncertainty to patients included the following key elements: (1) explaining the most likely as well as a brief differential diagnoses, (2) indicating what should be monitored (observation and next steps), (3) identifying the expected course for improvement and time frames, (4) acknowledging the limitations of the examination and tests, (5) facilitating access to clinicians, including contact information (when and how to call), and space and mechanism for patients to add thoughts and other input.

**Table 3.** Six Main Domains Incorporated Into Patient Leaflet for Communicating Diagnosis

General theme	Examples^a^	Source/study phase
Treatment plan, most likely/differential diagnoses	Our best thinking about diagnosis possibilities—the best and most important section; it hints at the doctor’s reasoning/logic regarding diagnosis.	PCP/phase 2
	Ideally, I would summarize and say based on the exam you [referring to the patient] have this, and we have not ruled out XYZ. Lay out the differential diagnosis to the part of making a plan.	PCP/phase 4
	Unlikely possibilities discussion is helpful, since this helps with unspoken fear.	
	Patients are typically given a diagnosis as 1 event and with no concern that it could be something else. It would be rare for a patient to say that I understood that beyond my diagnosis there is an issue.	Patient/phase 3
	I love the differential diagnosis, and the most likely and less likely bullets. If you have that you don’t have to have worries. For red flags you could put these are things that you should watch for.	Patient/phase 5
Observation and next steps for monitoring	What I have done over the years is going over a plan if you don’t get better. People always need a plan of what to do next.	PCP/phase 2
	You know, as next steps we’re going to, you know, maybe try this.	PCP/phase 4
	Most important thing would be that I liked walking away with all of my next steps written down and me looking at them on a piece of paper that is really helpful for me.	Patient/phase 5
	Patients could use the section beginning with next steps.	Patient/phase 3
Expected improvement and time frames	You have to realize [referring to the patient] that sometimes these symptoms go on for a long time and this is a shortcoming, but let us hope that the medications we give you are helpful and you will have a short recovery from the virus.	PCP/phase 4
	Good to include the following domain: should something arise after hours, you should contact: specific symptoms to watch for: to report immediately or to share with me at our next visit?	Patient/phase 3
Acknowledging examination and test limitations	We should explain that there are a lot of false-positives and this test entails a lot of radiation.	Clinician/phase 2
	I prefer it when the doctor explains why he did not order the test.	Patient/phase 5
Facilitating access with clinician’s contact information	It is great to keep it like this; calling the office and contact info are important. Letting them know the after [visit] contact information is good.	PCP/phase 4
	I especially like that they have the contact info here.	Patient/phase 5
Space to add patient thoughts and input	During the encounter with the patient or both after, it is nice if we both had a copy and wanted to write things down.	PCP/phase 4
	Include suggested patient questions and/or space to write down information, notes, questions.	Patient/phase 3

Abbreviation: PCP, primary care physician.

^a^ Selected illustrated quotations from PCP and patient interviews.

### Successive Versions of the Diagnostic Uncertainty Patient Leaflet/Tool

The initial leaflet (version I) was designed to be a comprehensive educational tool for explaining general information about diagnostic uncertainty and providing specific information for patients related to their presenting problem’s diagnostic assessment. In the 3 patient focus groups, views were expressed that it contained too much information and was not sufficiently focused on information relevant and desired by patients. This was noted and incorporated in subsequent versions (versions II-IV) (eAppendix 3 in Supplement 1). The final and most innovative revision consisted of a revised content and a workflow integration, enabling the leaflet to be produced during the visit via voice recognition dictation (eAppendix 3 in Supplement 1).

## Discussion

Managing diagnostic uncertainty and engaging in transparent conversations regarding uncertain diagnoses can promote a patient-centered approach, advance shared decision-making, and improve the reliability and safety of the follow-up.^30,31,32^ In this study built on these principles, PCPs and patients emphasized the importance of integrating diagnostic uncertainty in the clinical encounter conversations and workflow. Historically, clinicians often fail to discuss diagnostic uncertainty due to time constraints of clinical encounters, workloads, lack of experience, and discomfort in sharing their uncertainties.^33,34,35,36,37^ Prior studies rarely examined ways that may be effective for clinicians to share diagnostic uncertainty in a way that respects workflow constraints, captures the patient-clinician conversation, and is patient centered, including aiding patients in recall and engaging them in treatment decision-making. To our knowledge, this is the first study that has focused on developing guidelines for communicating diagnostic uncertainty to patients and investigating ways to practically integrate conversations about uncertainty into the workflow.

We developed suggestions for best practices to manage and communicate uncertainty and iteratively developed and implemented an innovative communication tool (eAppendix 3 in Supplement 1) for incorporating uncertainty language into a customizable patient leaflet to be captured via voice recognition technology during the encounter. The tool was developed based on the themes that emerged from the literature review, interviews with informatics experts and experienced PCPs (think-aloud scenario testing), and a series of focus groups and interviews with patients. It was designed to help clinicians manage diagnostic uncertainty, accurately document the encounter, and serve as a safety net for instructing patients on recommended testing, monitoring, and treatments. The clinicians using the tool during the clinical visit also found that it served as a forcing function to remind them to discuss uncertainties and address key communication domains. The PCPs implementing the tool were thus able to converse with patients while simultaneously dictating their diagnostic assessment via the voice-recognition software, producing a printable, patient-friendly, customized diagnosis handout for the patient. Although it was hoped this could also serve as the assessment portion of the clinical note (what one of the clinicians called “killing 3 birds with 1 stone,” namely, explaining the diagnosis to the patient verbally, producing a take-home leaflet, and populating the assessment for the note), the 2 clinicians were unable to make the tool work for this third function.

Recent increased attention to the importance of acknowledging diagnostic uncertainty is long overdue.^5,21,38,39,40^ While becoming more well recognized, diagnostic uncertainty needs to be practically, acceptably, and efficiently integrated in the clinical workflow. Enhancing verbal communication with a written patient leaflet is potentially valuable for meaningfully and reliably communicating uncertainty messages, a finding reinforced by our study, with patients strongly valuing the ability to take home verbally communicated messages. This can also be beneficial for patients with lower health literacy who may have difficulty comprehending verbal information. If translated to the patient’s primary language, the leaflet could serve as both a reminder and a guide to reference the information shared during the encounter. Even if available only in English, the leaflet may permit English-speaking patient family members to review it with the patient.

The 6 structural elements (building blocks) of the leaflet (explaining most likely/differential diagnoses; indicating what to monitor [observation and next steps]; identifying the expected course/improvement and time frames; acknowledging the limitations of examinations and tests; facilitating access to clinicians, including contact information [when and how to call]; and providing space/mechanism for patients to add thoughts and input) can provide practical guidance for developing interventions and training programs for health care professionals wishing to communicate diagnostic uncertainty, and could help less experienced clinicians who feel uncomfortable with discussing diagnostic uncertainty with patients.^40^ Less experienced clinicians could also benefit from such a tool to minimize diagnostic errors and earn patient trust.^41,42,43,44,45^ However, further research is needed to explore the different factors affecting clinician decisions to discuss diagnostic uncertainty, which may vary by patient, diagnosis, treatment timeline, workload, and time constraints, as well as clinician comfort level and patient preferences. We will need to learn more about best practices of incorporating such a tool into different types of encounters, such as emergency department visits.

### Limitations

This study has limitations associated with our ability to pilot test the diagnostic uncertainty communication tool. First, rather than a formal evaluation of a predefined implementation of the tool, this study was intended to iteratively develop the tool based on continuous feedback from a limited number of patients and clinicians. While the content of informational decision aids used in prior studies was generally predefined,^45,46,47^ in this study the leaflet had only the domains predefined, and the content was custom created during each encounter. Such open-ended design can cause content variability and challenge clinicians who need to perform the diagnostic assessments during the patient encounter, as opposed to, for example, composing these after the clinic visit. Second, the focus group patients participating in the 3 focus groups were different from average patients in that they were representatives of patient advocacy groups, and many of them had personally experienced diagnostic errors. This was both a strength (they had special insights) and a limitation (owing to their potentially unrepresentativeness; as one stated, “I want answers not excuses”) of our study. Third, we pilot tested the leaflet with a limited number of PCPs and patients, which limits the generalizability of our findings. In addition, the 2 PCPs who tested the tool were also involved in its development, which may have positively biased them. We tried to mitigate this bias through the evaluation by the communications researcher who independently collected and analyzed implementation of the patient feedback data. Initial plans for a larger participant sample were interrupted by the COVID-19 pandemic, resulting in termination of face-to-face encounters at the study site.

Regardless of these limitations, the 15 patients who assessed the tool universally and strongly voiced that they believed it was an improvement to their care and more than satisfactory in terms of information capture during conversations with clinicians. These caveats can be seen as opportunities to further refine and test the tool. Larger-scale testing, accounting for differences in work environments, diagnostic complexity, and clinical settings may be useful.

## Conclusions

Given the importance of discussing diagnosis and associated diagnostic uncertainties to patients and their clinicians, new approaches to facilitate such communication are warranted. Our pilot project helped map a framework for conveying key information around diagnostic uncertainty during the clinical encounter in the form of a written customizable summary. Clinicians and patients can benefit from such a structured tool when coproducing a diagnosis, and the tool may serve as the basis to systematically and proactively communicate diagnostic uncertainty.

## Notes

Supplement 1. **eAppendix 1.** Diagnostic Certainty Scenario Vignettes **eAppendix 2.** Clinicians’ Guide: Recommendations and Tips for Managing Uncertainty **eAppendix 3.** Versions I-IV of Patient Diagnostic Uncertainty Leaflet

Supplement 2.
Data Sharing Statement
