# Supplementary material for: Developing a Framework and Electronic Tool for Communicating Diagnostic Uncertainty in Primary Care: A Qualitative Study
Source: JAMA Netw Open. 2023 Mar 9;6(3):e232218. doi: 10.1001/jamanetworkopen.2023.2218 (PMC9999246; doi:10.1001/jamanetworkopen.2023.2218)
Supplement: Supplement 1. — eAppendix 1. Diagnostic Certainty Scenario Vignettes eAppendix 2. Clinicians’ Guide: Recommendations and Tips for Managing Uncertainty eAppendix 3. Versions I-IV of Patient Diagnostic Uncertainty Leaflet [file jamanetwopen-e232218-s001.pdf]

Supplementary Online Content

Khazen M, Mirica M, Carlile N, Groisser A, Schiff GD. Developing a framework and electronic tool for communicating diagnostic uncertainty in primary care: a qualitative study. *JAMA Netw Open*. 2023;6(3):e232218. doi:10.1001/jamanetworkopen.2023.2218

eAppendix 1. Diagnostic Certainty Scenario Vignettes

eAppendix 2. Clinicians' Guide: Recommendations and Tips for Managing Uncertainty

eAppendix 3. Versions I-IV of Patient Diagnostic Uncertainty Leaflet

This supplementary material has been provided by the authors to give readers additional information about their work.

© 2023 Khazen M et al. *JAMA Network Open.*

eAppendix 1. Diagnostic Certainty Scenario Vignettes

We are going to ask you to walk through four short patient scenarios to see how you might address uncertainty in these cases. But before we do, we'd like to ask you if you have any general thoughts or comments on your approach to communicating uncertainty with patients?

1) Guided discussion regarding the following cases:
   a. Lymphadenopathy in a patient with a history of non-Hodgkins lymphoma
      i. *40-year-old male with a history of non-Hodgkins lymphoma, apparently successfully treated and in remission, presents with concern about a new right neck mass. On exam, the mass is 2 cm and firm, and somewhat worrisome to the doctor and the patient. The patient is worried it could represent recurrence of the NHL; the physician is also concerned about this possibility.*
      ii. How would you discuss what you think this could be, and the uncertainties you have with the patient about his diagnosis?
   b. New headache in a middle-aged woman
      i. 56-year-old F presents with a new headache, with features most suggestive of a muscle tension headache, but the patient is worried that is could be something more serious. She asks if you're 100% certain it's not something else.
      ii. How would you discuss what you think this could be, and the uncertainties you have with the patient about her diagnosis?
   c. Cough, fever, chills in an otherwise healthy patient
      i. *A previously healthy 25-year-old presents with acute onset of cough, fever, and chills during the flu season. She is visibly distressed and wants to know if she could have antibiotics.*
      ii. How would you discuss what you think this could be, and the uncertainties you have with the patient about her diagnosis?
   d. Subacute low back pain
      i. *A 65-year-old man with a history of back pain that began several months ago after he missed a step and has been intermittently painful. He has tried ibuprofen and naproxen to little relief.*
      ii. **How would you discuss what you think this could be, and the uncertainties you have with the patient about his diagnosis?**

© 2023 Khazen M et al. *JAMA Network Open.*

eAppendix 2: Clinicians' Guide: Recommendations and Tips for Managing Uncertainty

Guiding Principle I – Validate patients experience and symptoms

- Acknowledge the impact of symptoms
    - "I'm glad you brought this up/came into the clinic"
- Elicit and acknowledge explanatory model
- Ask whether and/or why the patient is worried
- Invite patient to be an equal partner
- Align self with the patient:
    - "We are in this together"
    - "I will continue to be here for you"

Guiding Principle II – Be transparent about uncertainty

- "I believe that something is going on, but I do not yet know what it is."
- "Sometimes we don't have all the answers, but we will keep trying to figure out what is going on."
- Discuss limitations of modern medicine (testing)
    - Use stories to demonstrate uncertainty
    - "Hypotheses" rather than "diagnoses"
- Explain what about the symptoms are worrisome/not worrisome
    - Provide reassurance/concern where appropriate

Guiding Principles III - Create a Concrete Plan

- Plan for potential changes in symptoms
    - "Safety netting"
- Explain why time itself is a useful test
- Provide rationale for the treatment plan, prioritization
- Emphasize the positive, e.g., strategies to alleviate pain, percent of people who do well (not poorly)
- Give choices for treatments and reach consensus when possible
- Ensure the patient understanding
- Give contact information and be available
- Make clear you are open to changing your mind

Guiding Principles IV – Practices/Language to Avoid

- Don't assume the patient's concerns/worries– always best to ask
- Don't overwhelm with a laundry list of complications or possibilities
    - Avoid "nocebo" phenomenon
- Don't diminish symptoms
    - "It's all in your head"
- Don't refer to specialists or testing without explaining why

© 2023 Khazen M et al. *JAMA Network Open.*

eAppendix 3: Versions I – IV of Patient Diagnostic Uncertainty Leaflet

Patient Leaflet Version I

Getting to the Right Diagnosis – Working Together in the Face of Uncertainty

1. **Thank you for coming today**. We are glad you came in for us to assess your medical concerns and symptoms. If you are concerned, we are concerned. Thus, it is good you are here asking about your problem/symptom(s); we take every patient and every problem seriously. While we strive to give you the best explanation/diagnosis of what is wrong with you (so we can recommend the best treatment(s)), we often treat patients not being certain of the exact cause.

2. **Diagnosis is not a 100% exact science**. At times we don't get the exact right diagnosis immediately, or people often get better and we may never know the exact cause. Sometimes we just can't tell from the early symptoms that are nonspecific or atypical what the final diagnosis will turn out to be.
Tests (blood tests, x-rays) which can at times help sort out patients' diagnosis, don't always give a an exact answer or even can confuse or mislead us by giving false positive or false negative results (or even find abnormalities that don't matter for patients' health). Since every patient is different, and diagnoses are not always black or white or there can be multiple factors causing an illness, we usually have to consider a variety of possibilities and sometimes we are less certain than others about the diagnosis.

3. **Our best thinking about your diagnosis possibilities**. This is what is our best thinking of what is going on with you at this point, our best hypothesis/hypotheses of the diagnosis and cause of your problems/symptoms. We think that the most likely explanation of your problem/symptoms is

- ____

But there may be other possible causes (what we in medicine call a "Differential Diagnosis") such as:

- ____possible-unlikely-very unlikely
- ____possible-unlikely-very unlikely
- ____possible-unlikely-very unlikely

While this is not a complete list it can give you the flavor of other possibilities we might consider, especially if you do not improve or respond to treatment, or the illness evolves in unexpected ways.

4. **Your and Our worries**. It is important for us to hear/know what are your worries, and for you to hear what are some of the rarer but important possibilities that we want to keep in mind. This will help us know what to keep a watch for—what "red flags" that may signal we are wrong, or not addressing your symptoms as they change over time. Sometimes we refer to these worse-case scenario, not to worry you but instead so we can make sure we don't miss anything or unnecessarily delay an important diagnosis. Mostly, however we are here to **provide reassurance** that your problem is **not** something serious, because most symptoms turn out to benign, improve over time, or are diagnosis we can confidently say you don't have.

5. **Next steps**. The best way to get to the right diagnosis is usually to carefully follow patients over time to see how they do. In rarer cases where we are more worried, we will try to expedite additional testing and referrals, so we can have an answer as quickly as possible.

© 2023 Khazen M et al. *JAMA Network Open.*

To work out the best plan that we can all agree on, we want to create a concrete set of next steps that we all feel comfortable with:

We want to ensure you have easy access and are able to contact us if any questions or concerns arise. The best way(s) to reach me and our office is: ____

More specifically, to ensure good follow-up especially if you're not getting better or getting worse we will plan to do the following things together:

Scheduled follow up appt: ___________________________

Specific symptoms to watch for: ________________________

Time frame for expected improvement: _______________________________

Additional tests/specialist referral to consider in future: ___________________

6. **Working Together**. Jointly we have to be actively involved in better communication to get to the best possible diagnosis and patient outcomes. We are always trying to do better and learn from our patients and any mistakes. Since were in this together, we welcome your help to make us better diagnosticians and improve diagnosis overall. We urge you to review our visit notes (in Partners we have Open Notes in the patient Gateway portal) to refresh your memory and/or feedback to on what the doctor wrote.

© 2023 Khazen M et al. *JAMA Network Open.*

Patient Leaflet Version II

<table>
<tr><td colspan="2">Explaining the diagnosis by Working Together</td></tr>
<tr><td>Thank you for coming today. I take every patient and every problem seriously. While I strive to give you the best explanation/diagnosis for your problem, sometimes and in some cases I might treat patients not being certain of the exact cause.</td><td>Thank you for seeing me today.
I came because I evaluate your medical opinion and I trust that you will do your best to understand my problem and help me to resolve it.</td></tr>
<tr><td>Understanding the Diagnosis:
Diagnosis is not a 100% exact science. At times we (physicians) don't get the exact right diagnosis immediately. Sometimes we can't tell from the early symptoms what the final diagnosis will turn out to be. There can be multiple factors causing an illness, we usually have to consider a variety of possibilities and sometimes we are less certain about the diagnosis.</td><td>Comments and Thoughts:
____
____
____
____________________</td></tr>
<tr><td>Tests (blood tests, x-rays) which can at times help sort out patients' diagnosis, don't always give an exact answer or can mislead us by giving false positive or false negative.</td><td>Should I have additional tests (blood tests, x-rays) including:
• ____________________
• ____________________</td></tr>
<tr><td>Primary Diagnosis: My best thinking about your diagnosis.
The most likely explanation of your problem/symptoms:
• ____________________
• ____________________
• ____________________</td><td rowspan="2">Comments and Thoughts:
• ____________________
• ____________________
• ____________________
• ____________________
• ____________________
• ____________________
• ____________________</td></tr>
<tr><td>Explaining the Diagnosis: Reasons for this preferred/chosen diagnosis:
• ____________________
• ____________________
• ____________________
• ____________________</td></tr>
<tr><td>Findings not included in the Diagnosis and Less important possibilities that can explain your symptoms/ problem:
• ____________________
• ____________________
• ____________________</td><td>I am interested/ not interested in getting more information about the following possibilities:
• ____________________
• ____________________
• ____________________</td></tr>
</table>

© 2023 Khazen M et al. *JAMA Network Open.*

Patient Leaflet Version III

Working Together to Reach A Diagnosis: What To Do and Why Do We (Patients and Doctors) Need to Do It?

<table>
<tr><th colspan="2">DOCTOR</th><th>PATIENT</th></tr>
<tr><td colspan="2">UNDERSTANDING DIAGNOSIS: Diagnosis is not a 100% exact science.
• Often there can be multiple factors causing an illness. Thus, we might consider a variety of possibilities and sometimes we are less certain.
• Tests (such as blood tests/x-rays) may not always provide an exact answer. That's why we need to work together to be certain we give you the most appropriate treatment.</td><td>What Do I need to know?
____
____
____
____
____________________</td></tr>
<tr><td>Symptoms:
• ______________
• ______________</td><td>Findings from tests relevant to diagnosis
• ______________
• ______________</td><td rowspan="2">Have the symptoms changed? Are there new symptoms since last visit? Describe:
____
____
____
____
____
____________________</td></tr>
<tr><td colspan="2">The most likely explanation(s) of your symptoms:
• ____________________
• ____________________
Other possibilities:
• ____________________
• ____________________</td></tr>
<tr><td colspan="2">NEXT STEPS: Working together for most certain diagnosis and best treatment plan:
1. Schedule a follow-up visit by __________ (MM/DD/YY).
2. Watch for any of the following symptoms that may change over time and concern you.
• ____________________
3. Consider additional tests/specialist referral(s) in the future:
• ____________________
4. Keeping in touch: If any questions or concerns arise we encourage you to call us:
• ____________________</td><td>What Do I need to do when I get home? What is my Homework to get better?
____
____
____
____
____________________</td></tr>
<tr><td colspan="2">TIME FRAME FOR EXPECTED IMPROVEMENT:
_______Day(s); _____ week(s) ; ________ month(s); _________unknown</td><td></td></tr>
<tr><td colspan="3">I encourage you to review the visit notes. At Partners Healthcare you can access Open Notes in Patient Gateway portal.</td></tr>
</table>

© 2023 Khazen M et al. *JAMA Network Open.*

Version IV Tool as an End-of-Visit Voice Recognition Dictation Template

Figure 1. Version IV tool as an end-of-visit voice recognition dictation template.

ASSESSMENT/UNDERSTANDING YOUR DIAGNOSIS

**UNDERSTANDING The Diagnosis: It is rarely 100% certain**.<br>Often there can be multiple factors causing an illness. That is why doctors might consider a variety of possibilities for your diagnosis.<br>Tests (such as blood tests/x-rays) may not always provide an exact answer. That is why doctors and patients need to work together to provide the most appropriate treatment.	
**Symptoms**<br>• ***<br>• ***	**Findings from your tests relevant to diagnosis**<br>• ***<br>• ***
**The most likely explanation (diagnosis) of your symptoms (and my reasoning):**<br>• ***<br>***Other possibilities***:<br>• ***	
**NEXT STEPS:** Working together for most certain explanation and best treatment plan:<br>1. **Consider additional tests/specialist referral**(s) in the future:<br>***<br>2. **Schedule a follow-up visit** by: *** (x days/week)<br>3. **Watch for** any of the following symptoms that may change over time and concern you.<br>• ***<br>4. **Keeping in touch**: If any questions or concerns arise, we encourage you to call us. Office Pager Prefilled	
**TIME FRAME FOR EXPECTED IMPROVEMENT:**<br>_______Day(s); _____ week(s) ; ________ month(s); _________unknown	
**Have the symptoms changed? Are there new symptoms since last visit?**<br>***	
I encourage you to review the visit notes. At Partners Healthcare you can access **Open Notes** in Patient Gateway portal.	

Version IV is a replication of the Epic Dragon One Template. Each instance of 3 asterisks is a placeholder for dictated text that the clinician using a handheld Nuance Power Mic dictation microphone successively navigates through (via speech and the “next” button on the microphone) to enter customized text that fills in the patient leaflet.

An example of an actual completed leaflet, scrubbed of identifiable information, is shown below.

© 2023 Khazen M et al. *JAMA Network Open.*

<u>Patient Leaflet Version IV -Actual</u> leaflet example (anonymized)

Examples of leaflet IV tested in clinic

ASSESSMENT/UNDERSTANDING DIAGNOSIS

UNDERSTANDING The Diagnosis: It is not a 100% certain. Often there can be multiple factors causing an illness. That is why doctors might consider a variety of possibilities for treatment. Tests (such as blood tests/x-rays) may not always provide an exact answer. That is why doctors and patients need to work together to provide the most appropriate treatment.

Symptoms:

- **You are having some numbness in your left arm from your elbow forward not quite extending to your hand, you not have any weakness with it you noticed this when you are sleeping and sometimes when you are driving. You do not have any neck pain not have any similar symptoms in your other side and you not have any pain weakness in her hands. Not been have any fevers or chills. You do have some pain occasionally in your thumb but it does not seem related to this other symptoms.**

Findings from tests relevant to diagnosis

- **We did a physical exam today on the exam he did not seem to have any tenderness of the elbow joint there was no instability there was no mass there is no effusion your hand seemed normal your pulses were intact your sensation was intact and your shoulder seemed fine there was no pain when I pressed on your neck.**

The most likely explanation (diagnosis) of your symptoms:

- **Is a very common course for numbness shooting just from the elbow forward would be things related to the ulnar nerve the ulnar nerve travels through a small area at the bottom of the elbow. If you keep your elbow bent while sleeping that can cause some impingement because the nerve has to travel slightly longer distance and that can cause irritation to the nerve resulting in some numbness, or tingling. This can also occur when you have your arm raised and you have in the fixed position for period of time. The main treatment for this is to just make sure that you are adjusting your arm if you are keeping a single position and then when you are sleeping that you are not keeping it in a bent position. Sometimes people can do this just by thinking about it other times, other things you can do is to wrap it in a towel which will help you to stop it from bending quite a bit.**

Other possibilities:

- Other possibilities that it could be the nerves travel through the neck out to the arm and if there is some impingement in the cervical spine then you can have symptoms that sort of are in the distal distal part of the arm we often think of them is traveling across the arm and we often think about it extending across joint lines and so at this time I think is probably most likely that some things happen just at the elbow joint for you. Can be vascular causes that can be infectious causes those seem less likely since you otherwise have no other symptoms. It does not seem to be carpal tunnel syndrome or tennis elbow in which you have shooting pain on the ulnar side. Seem to be doing specific activities to be causing a golfer elbow either those are epicondylitis but inflammation on either side of the elbow. Since you do not have pain or symptoms there I do not think it is related to that. Thyroid conditions are sometimes associated with neuropathies, B12 deficiencies or others the neuropathies we often think affecting the longer nerves first which of those going to the feet less likely starting in the hands. But that something we can keep an eye on. You had normal thyroid function in the past and you have no known diabetes.

NEXT STEPS: Working together for most certain explanation and best treatment plan:

1. **Consider additional tests/specialist referral**(s) in the future: If it is persistent and unclear in the future we may consider doing some imaging of the neck if necessary or we can do EMG testing with a test the muscles of the hand.
2. **Schedule a follow-up visit** by: If you have persistent symptoms that are lasting more than 6 weeks then I would like to see you again. If at any time it suddenly worsens I would like to see you again.
3. **Watch for** any of the following symptoms that may change over time and concern you if you start noticing any of the symptoms in your feet or other hand if you notice any weakness or you have difficulty dropping things it becomes more severe or persistent.
4. **Keeping in touch**: If any questions or concerns arise, we encourage you to call us. The clinic number is [redacted]

I encourage you to review the visit notes. At Partners Healthcare you can access **Open Notes** in Patient Gateway portal.

© 2023 Khazen M et al. *JAMA Network Open.*
