# Supplementary material for: Developing a Framework and Electronic Tool for Communicating Diagnostic Uncertainty in Primary Care: A Qualitative Study
Source: JAMA Netw Open. 2023 Mar 9;6(3):e232218. doi: 10.1001/jamanetworkopen.2023.2218 (PMC9999246; doi:10.1001/jamanetworkopen.2023.2218)
Supplement: Supplement 2. — Data Sharing Statement [file jamanetwopen-e232218-s002.pdf]

Data Sharing Statement

Khazen. Developing a Framework and Electronic Tool for Communicating Diagnostic Uncertainty in Primary Care. *JAMA Netw Open*. Published March 09, 2023. doi:10.1001/jamanetworkopen.2023.2218

Data
Data available: No

Additional Information
Explanation for why data not available: based on IRB protocol
